# Rethinking CRISPR delivery for liver-targeted gene editing: The case for spatially fractionated intra-arterial approaches

**DOI:** 10.1016/j.omtn.2025.102782

**Published:** 2025-12-01

**Authors:** Abdallah Salemdawod, Piotr Walczak, Miroslaw Janowski

**Affiliations:** 1 Program in Image Guided Neurointerventions, Department of Diagnostic Radiology and Nuclear Medicine, University of Maryland School of Medicine, Baltimore, MD, USA

## Main text

The recently reported death of a clinical trial participant treated with intravenous (i.v.) Intellia Therapeutics' nexiguran ziclumeran (nex-z) for transthyretin amyloidosis, marked by grade 4 liver enzyme elevations and increased bilirubin following lipid nanoparticle (LNP)-CRISPR delivery, underscores a fundamental limitation of systemic approaches: the inability to fractionate treatment when the entire liver is simultaneously exposed. This tragedy demands critical reassessment of delivery strategies for liver-directed gene editing, with intra-arterial delivery via hepatic artery infusion, offering a more controllable alternative that preserves hepatic reserve, lowers peak exposures, and allows adaptive dosing across sessions, while maintaining or even improving intrahepatic bioavailability.

### The systemic delivery dilemma

LNP-based CRISPR delivery has achieved remarkable preclinical efficacy, with single i.v. administrations producing >97% reductions in target protein levels.^1^ However, this systemic approach presents inherent vulnerabilities. LNPs exhibit natural hepatotropism through apolipoprotein E-mediated (ApoE-LDLR) uptake, resulting in broad parenchymal exposure following a single i.v. dose. This creates an all-or-nothing scenario where therapeutic agents bathe the entire organ simultaneously, leaving no hepatic reserve to buffer if severe toxicity develops.

The mechanisms underlying LNP-mediated hepatotoxicity involve multiple pathways. Ionizable lipids within LNPs accumulate in liver sinusoidal endothelial cells, triggering activation and induction of neutrophil-related cytokines, followed by neutrophilic inflammation.^2^ This hepatotoxicity cascade can progress rapidly, and recent safety data demonstrate that even biodegradable LNP formulations blunt but do not abolish dose-linked signals and the steepness of the dose-toxicity curve becomes especially perilous when the entire organ is simultaneously perfused.^3^ When the entire hepatic mass receives simultaneous high-dose exposure, individual variability in immune responses or underlying disease can prove catastrophic, as demonstrated by the Intellia case.

### Fractionated intra-arterial delivery: a paradigm shift

Hepatic artery infusion reframes liver gene editing as a regional, iterative procedure, enabling sequential treatment of discrete liver segments while maintaining functional hepatic reserve. By treating only a portion of the liver during each session (e.g., right lobe segments in one procedure, left lobe in another), the total single-session LNP dose can be reduced to 25%–40% of the systemic i.v. equivalent while achieving comprehensive organ coverage over 3–4 treatment cycles spaced 2–4 weeks apart.^4^

This spatiotemporal fractionation strategy yields several practical benefits. First, lower peak LNP concentrations per session reduce acute inflammatory responses. Second, inter-session clearance time allows monitoring for early toxicity signals. Third, and most critically, treatment can be halted if complications emerge, preserving untreated hepatic tissue. The hepatic artery infusion infrastructure is well-established, with Food and Drug Administration-approved implantable pump systems demonstrating 94.9% stability at 6 months and technical success rates exceeding 94% in experienced centers.^5^

**Figure 1:**
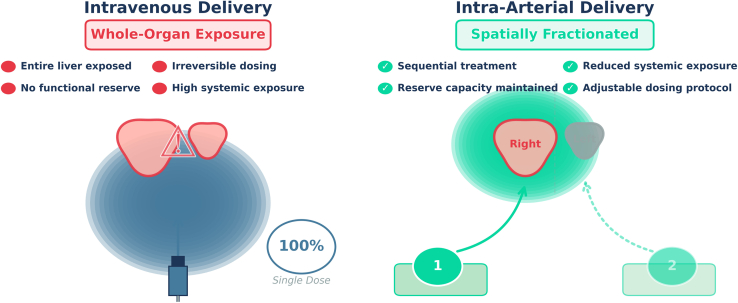
Visual representation of a concept of spatially fractionated intra-arterial delivery, in comparison to traditional whole-organ, systemic delivery

The pharmacokinetic advantages are substantial. Hepatic arterial delivery achieves 40- to 120-fold higher concentrations in perfused versus non-perfused liver lobes in preclinical models, with first-pass hepatic extraction limiting systemic exposure.^6^ For gene delivery applications, selective regional delivery via hepatic artery achieves dramatically enhanced transduction efficiency compared to i.v. routes while reducing off-target organ exposure.^7^ This therapeutic separation prioritizes safety without sacrificing coverage, which is precisely what CRISPR liver therapeutics require (Figure 1).

### Enabling technologies: MRI guidance and osmotic enhancement

Modern interventional magnetic resonance imaging (MRI) enables radiation-free, real-time visualization of catheter positioning and confirms homogeneous therapeutic distribution, a critical safety feature absent from conventional approaches. MRI’s superior soft tissue contrast allows dynamic monitoring of perfusion patterns during delivery, with immediate verification of proper arterial flow. Preclinical models demonstrate reproducible hepatic artery catheterization with 94.1% technical success when performed under imaging guidance.^8^

Incorporation of osmotic blood-organ barrier disruption protocols further enhances regional delivery. Intra-arterial infusion of 25% mannitol and 4% hypertonic saline induces transient endothelial cell shrinkage through osmotic gradient establishment improving biological agent penetration for a reversible window.^9^ This technique produces 10-fold increases in macromolecule uptake with reversible disruption. Applied to hepatic delivery under MRI guidance, osmotic enhancement could enable dramatic improvements in LNP cellular uptake, potentially allowing for significant dose reductions while maintaining therapeutic editing efficiency, or conversely enable deeper editing at the same dose.^10^

### CRISPR packaging

LNPs have become the dominant platform for packaging CRISPR-Cas9 systems, delivering the nuclease as mRNA. This approach evolved from the safety requirements of mass vaccination campaigns and leverages the inherent hepatic tropism of LNPs. However, other Cas9 formats may be superior for broader gene editing applications. First, delivering Cas9 as a protein enables immediate activity compared to mRNA-based approaches, as it bypasses the need for intracellular transcription and translation. Importantly, Cas9 protein can be safely and effectively delivered to cells using simple lipid-based transfection reagents such as Lipofectamine.^11^ When combined with intra-arterial delivery, these akin-to-bench-scale lipoplexes have demonstrated outstanding *in vivo* efficacy of cargo delivery.^12^ Therefore, the intra-arterial route may offer greater flexibility in selecting both the form of Cas9 (protein versus mRNA) and the packaging system itself, which enables tuning chemistry for biocompatibility over brute-force systemic exposure.

### Clinical precedent and path forward

Extensive clinical experience with hepatic artery infusion chemotherapy provides robust safety data supporting this approach.^13^ Hepatic arterial infusion in conjunction with systemic chemotherapy augments response rates to 85%–92% in patients with liver-dominant disease, with biliary toxicity (manageable with corticosteroid prophylaxis) representing the primary concern, while systemic toxicity is largely avoided.^4^ This therapeutic separation validates the principle of regional delivery.

The path forward requires rigorous preclinical validation in large animal models comparing i.v. versus intra-arterial LNP-CRISPR delivery, with comprehensive assessment of biodistribution, editing efficiency, inflammatory responses, and dose-response relationships. Early-phase clinical trials should evaluate fractionated regional delivery protocols in liver-specific genetic disorders. The goal is not merely to replicate systemic delivery through a different vascular route, but to fundamentally reconceptualize liver gene editing as a regional, iterative process, treating the organ not as a monolithic target but as a collection of addressable territories that can be modified safely over time.

For patients like the one lost in the Intellia MAGNITUDE trial, this distinction could prove lifesaving. The field of *in vivo* gene editing stands at a critical juncture. While systemic approaches have demonstrated proof-of-concept efficacy, the inability to fractionate treatment and the steep dose-toxicity relationship create unacceptable risks when targeting an essential organ. Intra-arterial delivery via hepatic artery infusion, implemented through fractionated protocols with MRI guidance and optional osmotic enhancement, addresses these limitations directly, while leveraging decades of established interventional experience. Recent advances in miniaturization of soft robotic tools for endovascular interventions are expanding the capabilities of intra-arterial drug delivery.^14^

### Conclusion

The i.v. LNP-CRISPR made history by proving that *in vivo* gene editing is possible in humans. But proof of concept is not the endpoint. The steep dose-toxicity relationship, organ-wide exposure, and inability to fractionate risk argue for a shift to fractionated, image-guided intra-arterial delivery. By lowering per-session dose, preserving hepatic reserve, and enabling feedback-driven adaptation, intra-arterial route can raise the ceiling on safety while sustaining or improving efficacy. With MRI guidance and optional osmotic priming, we can further sharpen regional pharmacology. And by revisiting the payload chemistry (including ribonucleoproteins), we can align editing pharmacodynamics with a controllable delivery backbone. In doing so, we honor both the promise of gene editing and the obligation to deliver it as safely and thoughtfully as possible.

## Acknowledgments

Maryland Stem Cell Research Fund: 2024-MSCRFD-6430.

## Declaration of interests

M.J. and P.W.P. are co-founders of IntraART and Ti-com, which are not related to the current submission. P.W. and M.J. have also patented in the area of endovascular procedures, and P.W.P., M.J., and A.S. have also patent on genome editing.
